# Overcoming air-water interface-induced artifacts in Cryo-EM with protein nanocrates

**DOI:** 10.1101/2025.08.18.667046

**Published:** 2025-08-18

**Authors:** Matthew C Jenkins, Daija Bobe, Jake D Johnston, Jonah Cheung, Akira Karasawa, Christina M Zimanyi, Ömer Dermanci, M.G. Finn, Alex de Marco, Mykhailo Kopylov

**Affiliations:** 1 School of Chemistry and Biochemistry, School of Biological Sciences, Georgia Institute of Technology, Atlanta, GA, 30332, USA; 2 Simons Electron Microscopy Center, New York Structural Biology Center, New York, NY, 10027, USA; 3 Department of Physiology and Cellular Biophysics, Columbia University, New York, NY, 10027, USA

## Abstract

Contact with the air-water interface can bias the orientation of macromolecules during cryo-EM sample preparation, leading to uneven sample distribution, preferred orientation, and damage to the molecules of interest. To prevent this, we describe a method to encapsulate target proteins within highly hydrophilic, structurally homogeneous, and stable protein shells, which we refer to as “nanocrates” for this purpose. Here, we describe packaging, data acquisition, and reconstruction of three proof-of-principle examples, each illuminating a different aspect of the method: apoferritin (ApoF, demonstrating high-resolution), thyroglobulin (Tg, solving a known preferred orientation problem), and 7,8-dihydroneopterin aldolase (DHNA, a structure previously uncharacterized by cryo-EM).

Cryo-electron microscopy (cryo-EM) can provide high-resolution structural information about biomolecules in vitreous ice. Resolving such three-dimensional reconstructions requires averaging of tens to hundreds of thousands of randomly oriented individual particles. However, biomolecules frequently concentrate at the air-water interface (AWI) during sample preparation, often adopting preferred orientations that make three-dimensional reconstruction difficult or impossible due to insufficient orientation sampling.^1,2^ Several methods have been developed to address this issue,^3–5^ including sample tilting during data collection,^6^ modifications to EM grids^7–9^ (often involving the addition of randomly-oriented binding interactions^10–12^), changes in sample preparation or freezing methods,^7,13–16^ and the use of detergents^17–19^ or barrier proteins.^20,21^ DNA superstructures have also been used to orient encapsulated or attached proteins for cryo-EM analysis, although for different purposes, resulting in reconstructions of moderate resolution.^22,23^ None of these methods offers a complete solution to AWI-related issues.

Our approach was inspired by the established use of natural virus capsids and engineered protein containers for packaging applications.^24–27^ We adapted this concept for cryo-EM by encapsulating target proteins within symmetric MS2 bacteriophage-derived “nanocrates”. Being highly symmetric, the nanocrates do not adopt a preferred orientation at the AWI, and therefore their enveloped cargoes are randomly oriented. Nanocrate densities can be computationally subtracted during single-particle cryo-EM analysis to allow for high-resolution reconstruction of the molecules inside.

MS2 virus-like particles have multiple properties that make them ideal for use as nanocrates. They are expressed and isolated in high yields and are amenable to controlled disassembly and reassembly around cargo proteins by a simple pH-dependent protocol.^28–30^ Disassembled MS2 coat proteins can be stored at 4°C for at least a day and at −80°C for several months without losing reassembly and encapsulation efficiency. At 21 nm, the interior diameter of the MS2 particle is large enough to accommodate many target cargoes.^31^ Importantly, the MS2 nanocrates themselves are highly monodisperse (97% of wild-type MS2 particles are T=3 icosahedra),^31,32^ which is a requirement for the signal subtraction step needed to reconstruct cargoes.^33–35^ As expected, packaged cargo molecules are randomly oriented with respect to the cryo-EM grid, enabling high-resolution isotropic reconstructions, as demonstrated here for three proteins at resolutions of 2.1–2.9 Å.

We first packaged apoferritin (ApoF)—a homo 24-mer of 484 kDa total molecular weight, often used as a standard cryo-EM high-resolution benchmark (Figure 1A). Briefly, we purified MS2 capsids from *E. coli*, performed disassembly in an acidic solvent with the removal of packaged viral RNA by centrifugation, and reassembled the capsid proteins at neutral pH in the presence of ApoF. We designate the reassembled cages as nanocrates (“nc”) to distinguish particles derived from disassembly and reassembly, rather than another commonly used packing strategy in which cargo-containing virus-like particles (VLPs) are derived from simultaneous expression and packaging in the expression host.^24,36,37^ The @ symbol is used to designate the packaged species. After reassembly, the reaction was concentrated 10-fold, vitrified on cryo-EM grids, and imaged on a Thermo Fisher Scientific Titan Krios microscope equipped with a Gatan K3 camera.

Cryo-EM image analysis of MS2nc@ApoF reassembly showed a mixed population of empty and cargo-filled MS2nc particles (Figure 1B). From 5,000 micrographs, we selected ~240,000 particles and used icosahedral symmetry to produce a map of MS2nc at a resolution of 1.86 Å. Computational “particle subtraction” from cargo-containing 2D images revealed the ApoF inside, and standard single-particle analysis of the subtracted images yielded a 2.16 Å resolution structure of ApoF (Figures 1C, S4).

This level of resolution for ApoF is lower than the sub-2 Å resolution routinely achieved for the protein alone. This difference may stem from imperfect particle subtraction or a higher background due to the need for effectively thicker ice compared to what could be achieved with isolated proteins that are smaller than MS2nc. More sophisticated methods for accurate particle subtraction are being developed^38^ that may further improve resolution, though current approaches already deliver structures suitable for detailed molecular analysis. Importantly, the structure of nanocrate-packaged ApoF is indistinguishable from structures determined by conventional methods,^39,40^ demonstrating that the nanocrate environment does not induce conformational changes.

Having established that nanocrates can successfully encapsulate ApoF and that our proposed processing pipeline yields high-resolution reconstructions, we next tested the applicability of nanocrates with bovine thyroglobulin (Tg), a protein with well-documented preferred orientation problems in conventional cryo-EM. Previous high-resolution structures of Tg required detergents or other specialized procedures.^41–46^

Packaging Tg into MS2 nanocrates presented a challenge: the protein’s dimensions exceed the available inner diameter of the MS2 icosahedral shell. Rather than preventing encapsulation, this resulted in one lobe of Tg protruding through the MS2 shell (Figure 1D). This partial encapsulation still provided adequate protection from air-water interface effects, yielding a 2.94 Å resolution reconstruction with no denatured Tg particles observed in 2D classification.

The presence of both packaged and free thyroglobulin in the nanocrate preparation allowed for a direct comparison, revealing substantial advantages provided by encapsulation. Analysis of orientation distributions revealed that Tg from nanocrates achieved better angular sampling than non-encapsulated Tg from the same dataset (Figure 2A, 2B). More importantly, the nanocrate-derived structure achieved higher resolution (3.4 Å with C1 symmetry) compared to the structure (3.9 Å with C1 symmetry) obtained from equivalent numbers of free protein images, demonstrating that improved orientation distribution directly translates to better structural information.

The Tg reconstruction revealed that the protein remained highly dynamic even within nanocrates, as evidenced by weaker density at distal regions (Figure S5C). Masked local refinement enabled better visualization of these dynamic regions (Figure S5D-F); however, full model building would require additional 3D classification and local filtering approaches.^41–46^ Thus, the use of nanocrates eliminated the preferred orientation artifacts that have historically complicated Tg structure determination, converting a problematic target into a routinely processable sample.

Finally, we applied MS2nc to the challenging cryo-EM target of 7,8-dihydroneopterin aldolase (DHNA), a barrel-shaped octameric protein assembly that adopts extreme preferred orientation on conventional grids. Our attempts to determine the DHNA structure by standard methods yielded highly anisotropic maps unsuitable for model building, despite achieving a GSFSC resolution of 2.29 Å (Figure S7). The orientation distribution plots revealed the severity of the problem: DHNA particles adopted essentially identical orientations, providing insufficient angular sampling for meaningful 3D reconstruction (Figures 2C and 2D). Since DHNA has an assembled molecular weight of 107 kDa and an expected diameter of 70 Å, we anticipated it would be readily packaged in MS2nc.

We observed MS2nc@DHNA particles containing multiple copies of DHNA in an ordered C5 symmetric arrangement (Figure S6C), allowing the use of a symmetry expansion workflow to obtain the final 3D reconstruction (Figures S6F). MS2nc@DHNA yielded a 2.80 Å resolution reconstruction (Figure 1E) with well-distributed particle orientations (Figure 2D) making it suitable for atomic model building.

From a methodological perspective, we found that one could not simply use the published structure of MS2 for the nanocrate density subtraction step. Instead, it was necessary to employ the experimental data for MS2nc obtained for each sample to calculate the shell density for subtraction. The nanocrate thereby provides a built-in resolution standard, determining the principal resolution limit of the dataset. It can also be used to tune dataset processing parameters such as coma, magnification anisotropy, per-particle CTF, Cs value, and pixel size.

In summary, nanocrates offer significant practical advantages for the high-resolution reconstruction of proteins by single-particle cryo-EM. The method improved the angular orientation of particles that suffered from preferred orientations by traditional plunge freezing methods. Using the same protocol, we observed significantly different encapsulation rates for the various cargos: 25% for MS2nc@ApoF, 30% for MS2nc@Tg, and 65% for MS2nc@DHNA. It proved unnecessary to modify the cargo loading protocol for each sample, as these variations in the extent of cargo loading did not hinder resolution as measured by gold-standard Fourier-shell correlation. Furthermore, unlike conventional methods requiring extensive optimization of grid preparation parameters, nanocrates enable standardized cryo-EM preparation since their consistent external surface properties, regardless of cargo, govern particle behavior during plunge freezing. This removes the need to re-optimize sample concentration, grid type, and blotting methods, providing significant time and cost savings while reducing consumption of expensive samples, consumables, and microscope time. The use of nanocrates also eliminates air-water interface effects through complete molecular shielding. While we expect that protein-cage interactions may occasionally cause problems, it should be possible to optimize the properties of the interior particle surface for specific specimens or to employ other self-assembling nanoparticle containers as nanocrates.

## Methods

### MS2 capsids expression and purification

MS2 capsids can be purified, disassembled, and reassembled by various methods,^29,30,47–49^ and the optimal protocol will depend on the equipment and standard procedures of each laboratory. In supplemental methods, we provide two such protocols – one from the Georgia Tech laboratory and another from NYSBC. Both provided approximately the same yield and quality of MS2 capsids: typically, 10–50 mg of purified MS2 VLPs were isolated from 1 L cell culture.

### Disassembly of MS2 VLPs

For simultaneous capsid disassembly and encapsulated RNA removal, 1 mL of glacial acetic acid was added dropwise over ~30 seconds (without stirring) into a 0.5 mL solution of MS2 capsids (10 mg/mL) in 1x PBS. The solution was capped, mixed by inversion 2–3 times, and then incubated on ice for 20 minutes. The cloudy solution was then centrifuged at 6,600g for 10 minutes at 4°C to pellet out precipitated RNAs and any stochastically precipitated MS2 coat proteins. The clear supernatant, containing soluble disassembled MS2 coat protein dimers, was then removed by aspiration and loaded into a NAP-25 column (Cytiva) that had been pre-equilibrated with aqueous 1 mM acetic acid. MS2 dimers were eluted in 10 × 0.5 mL fractions of 1 mM acetic acid, which were immediately placed on ice. The protein concentration of each elution fraction was determined using the absorbance value at 280 nm on a NanoDrop instrument (using the theoretical calculation of 1.235 absorbance units for 1 mg/mL concentration, as determined from the MS2 primary sequence using the ExPASy ProtParam online tool, https://web.expasy.org/protparam/).^50^ The 3–4 most concentrated fractions were pooled and either used immediately for cargo packaging reactions or stored at −80°C for future use.

### Cargo packaging within reassembling MS2 VLPs

Packaging of cargo proteins within reassembling MS2 VLPs was achieved by first mixing the cargo protein of interest, water, and a 1/10^th^ volume of 10x TMK buffer (0.1 M Tris-HCl [pH 8.5], 80 mM KCl, 10 mM MgCl_2_)^47,51^ relative to the intended final reassembly solution volume in a 1.5 mL Eppendorf tube. Disassembled MS2 coat protein dimers in 1 mM acetic acid were subsequently added to complete the reassembly mixture. The Eppendorf tube was then capped and gently agitated at room temperature (~25°C) with an end-over-end mixer for 3 hours. Following capsid reassembly, the sample mixture was stored at 4°C prior to shipping to NYSBC for cryo-EM imaging.

**Figure 1.**
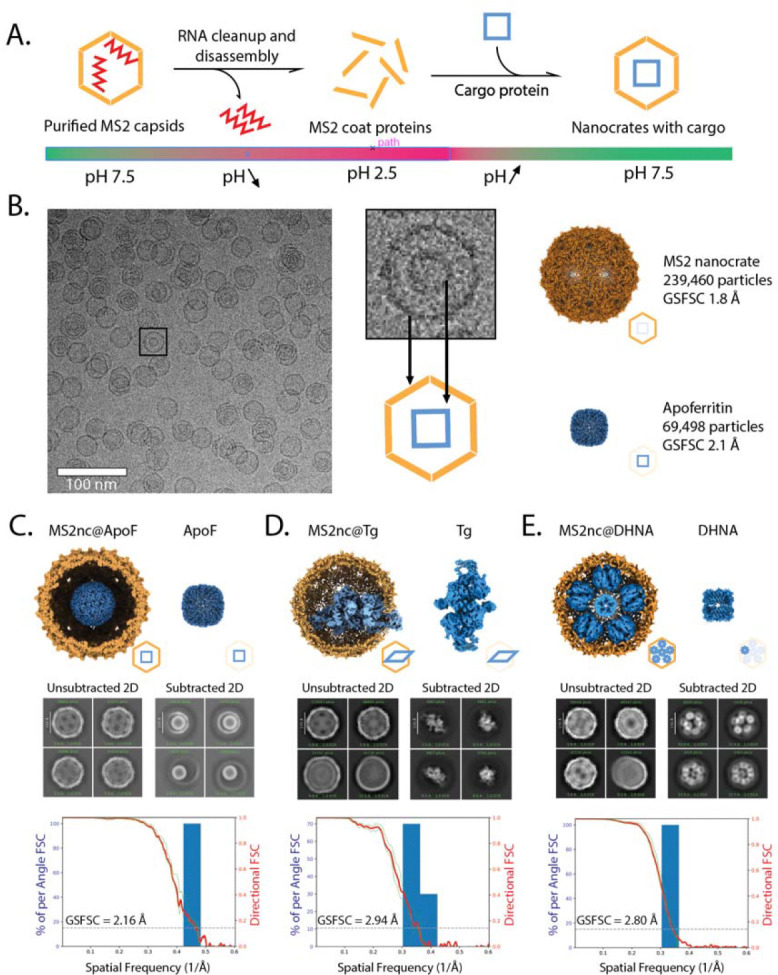
Structure determination of three proteins from nanocrates. **A.** Schematic representation of the nanocrate method as described in the text. B. Representative cryo-EM micrograph of MS2nc@ApoF particles, with a cropped out single particle showing the MS2 shell and encapsulated ApoF cargo and cryo-EM maps obtained after processing the MS2nc@ApoF dataset. The MS2nc was processed with icosahedral symmetry; after particle subtraction, ApoF was processed with octahedral symmetry. **C-E.** Summary of nanocrate-based structure determination for ApoF, Tg, and DHNA, respectively. (*Top*) Complete reconstructions, with cargo proteins in blue and MS2nc in yellow shown to the left; cargo-only reconstructions are shown on the right. (*Middle*) 2D class averages before and after nanocrate density subtraction. (*Bottom*) 3D FSC plots for each cargo protein.

**Figure 2.**
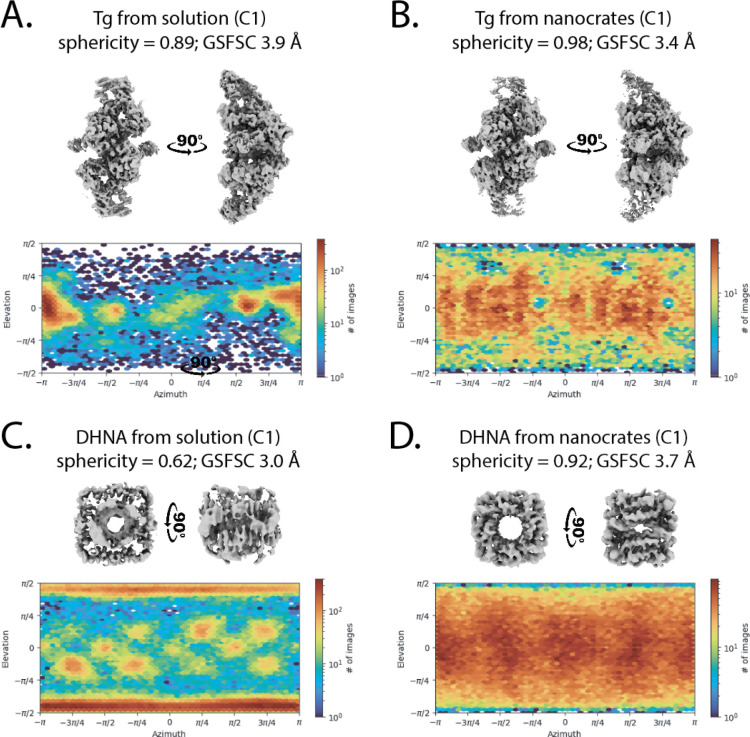
Nanocrate encapsulation eliminates preferred orientation. **A.** Cryo-EM map and orientation distribution plot of 32,904 Tg particles refined with C1 symmetry. **B.** Nanocrate-derived cryo-EM map and orientation distribution plot of 32,904 Tg particles refined with C1 symmetry. Note the improved resolution of the nanocrate-derived structure versus the solution map. Both reconstructions from **A** and **B** were derived from the same dataset, which contained both non-packed and MS2-packaged Tg molecules. **C.** Cryo-EM map and orientation distribution plot of 112,060 DHNA particles refined with C1 symmetry from the conventionally prepared cryo-EM grids. Despite relatively high GSFSC resolution, the map is unsuitable for model building due to low sphericity. D. Nanocrate-derived cryo-EM map and orientation distribution plot of 112,060 DHNA particles refined with C1 symmetry.

Several reassembly mixtures were prepared for each cargo molecule in which the final concentration of cargo protein monomers was varied in relation to a fixed concentration of MS2 coat protein monomers. In all cases, an abundance of MS2 coat proteins was included relative to cargo protein since each reassembling nanocrate requires 180 monomers to form a complete VLP shell. Consequently, the MS2 coat protein monomer to cargo monomer ratios (i.e., MS2:cargo ratios) were tested over a range of 12.5:1 up to 200:1 in nanocrate reassembly solutions. The specific MS2:cargo ratios used to collect the cryo-EM data presented in Figures 1 and 2 are included in Table S1. An example reassembly mixture is presented in Table S2.

### Negative staining and EM data acquisition

EM grids (Ted Pella Inc, Carbon Type-B copper 300 mesh) were plasma cleaned using a H_2_/O_2_ gas mixture for 30 s in a Solarus Plasma Cleaner 950 (Gatan). The VLP sample (3 μL, 0.1 mg/mL in PBS) was applied to the grid and allowed to adsorb for 30 s before blotting away excess liquid. This was followed by two cycles of washing with deionized (Milli-Q) water, blotting, and staining with 2% (w/v) uranyl acetate for 45 s before final blotting. Negatively stained grids were imaged using a Hitachi-7800 transmission electron microscope at an accelerating voltage of 100 keV, a nominal magnification of 120,000x (corresponding to a pixel size of 1.8 Å) and defocus ranging from −2.0 to −3.0 μm.

### Cryo-EM sample preparation and data acquisition

Unless otherwise specified, the MS2nc@cargo samples were concentrated 10-fold using spin concentrators with a 100 kDa molecular weight cut-off. The sample was applied to plasma-cleaned UltrAuFoil 1.2/1.3 grids (H_2_/O_2_ gas mixture for 7 s in a Solarus Plasma Cleaner 950 (Gatan)) and imaged on a TFS Titan Krios instrument G2 equipped with a Gatan K3 camera and a Gatan Bioquantum energy filter. Data were acquired using Leginon^52,53^ in counting mode with a calibrated pixel size of 0.832 Å and a 20 eV energy slit. The total dose was 55 e^−^/A^2^ per movie, split into 50 frames. Movie stacks were motion corrected and dose-weighted with motioncorr2^54^, and the resulting images were imported into CryoSPARC^55^ for processing.

### Data processing

Data processing was performed using standard SPA tools in two major steps. First, the dataset was processed to achieve high-resolution reconstruction of the nanocrate, followed by particle subtraction. Second, the subtracted stack was processed to reconstruct the cargo proteins. The overall processing workflow is summarized in Figure S2, and detailed descriptions of individual cargoes are provided in Supplemental Methods. Plots in 1C, 1D, 1E, and sphericity values were generated by processing final refinements using the 3DFSC server.^6^

## Supplementary Material

Supplement 1
