# Supplementary material for: Overcoming air-water interface-induced artifacts in Cryo-EM with protein nanocrates
Source: bioRxiv. 2025 Aug 18:2025.08.18.667046. Preprint. [Version 1] doi: 10.1101/2025.08.18.667046 (PMC12393331; doi:10.1101/2025.08.18.667046)
Supplement: Supplement 1 [file NIHPP2025.08.18.667046v1-supplement-1.pdf]

bioRxiv preprint doi: https://doi.org/10.1101/2025.08.18.667046; this version posted August 18, 2025. The copyright holder for this preprint (which was not certified by peer review) is the author/funder, who has granted bioRxiv a license to display the preprint in perpetuity. It is made available under aCC-BY 4.0 International license.

Supplemental methods:

<u>MS2 expression and purification – method #1 (Georgia Tech)</u>

The pCDF:MS2 plasmid encoding the MS2 coat protein gene was transformed into chemically competent BL21(DE3) *E. coli* cells (New England Biolabs), and positive transformants were selected by plating cells onto 2YT agar plates containing a final concentration of 50 µg/mL streptomycin. After overnight incubation at 37°C, transformant cells were gently scraped from a single colony with a sterile pipette tip and were inoculated into 500 mL of 2YT or SOB medium supplemented with 50 µg/mL streptomycin in a 2 L baffled Erlenmeyer flask. The culture flask was then grown to saturation by incubation at 37°C for 20-24 hours in a shaking incubator set to a rotational speed of 180 rpm. Cells were subsequently harvested from the culture volume by centrifugation at 8,000 rpm for 15 minutes at 4°C in a JLA-16.250 rotor (Beckman Coulter), and the resulting cell pellets were stored at -80°C until purification.

Purification of MS2 VLPs was performed by first resuspending cell pellets from an entire 0.5 L expression batch in 50 mL of 50 mM potassium phosphate buffer (pH 7.0). The resuspended cell slurry was transferred to a 100 mL glass beaker. The cells were lysed via sonication in an ice/water bath using a QSonica Q500 sonicator equipped with a 0.5" probe tip (5-second sonication pulses with 5-second rest between pulses at an instrument amplitude value between 45-55%, 120 pulses in total). The cell lysate was then clarified by centrifugation at 14,000 rpm for 15 minutes at 4°C in a JA-17 rotor (Beckman Coulter). VLPs were subsequently precipitated from the clarified lysate by adding solid ammonium sulfate to a final concentration of 0.265 g/mL, dissolving the ammonium sulfate solids at room temperature, and then incubating the mixture on an end-over-end mixer at 4°C for 1 hour. Precipitated solids were collected by centrifugation at 14,000 rpm for 15 minutes at 4°C in a JA-17 rotor and resuspended in 8 mL of fresh 50 mM potassium phosphate buffer (pH 7.0). Residual hydrophobic contaminants were removed by measuring the volume of the VLP solution (which usually expanded to 10-12 mL following solubilization of precipitated solids) and then adding an equal volume of an organic solution consisting of *n*-butanol and chloroform in a 1:1 ratio to the aqueous VLP solution. The aqueous/organic mixture was vigorously agitated for 1 minute, then the respective aqueous

bioRxiv preprint doi: https://doi.org/10.1101/2025.08.18.667046; this version posted August 18, 2025. The copyright holder for this preprint (which was not certified by peer review) is the author/funder, who has granted bioRxiv a license to display the preprint in perpetuity. It is made available under aCC-BY 4.0 International license.

and organic layers were resolved by centrifugation at 14,000 rpm for 10 minutes at 4°C in a JA-17 rotor. The upper aqueous layer was gently removed by aspiration and then layered on top of 10-40% sucrose gradients (~3 mL VLP solution per 40 mL gradient tube) prepared in 50 mM phosphate buffer (pH 7.0). Gradient sedimentation was performed by ultracentrifugation at 28,000 rpm for 4 hours at 4°C in a SW32 rotor (Beckman Coulter).

VLPs were visualized by bottom-up white light illumination of sucrose gradient tubes and were carefully removed by aspiration with a syringe. Isolated VLPs were then concentrated by ultracentrifugation at 68,000 rpm for 2 hours at 4°C in a Type 70 Ti rotor (Beckman Coulter), and the resulting supernatant was decanted. The pelleted VLPs in each ultracentrifuge tube were gently resuspended in 3-5 mL of fresh 1x PBS buffer, sterile filtered through a 0.2 µm PES syringe filter, and stored at 4°C (short-term) or -80°C (long-term). Before storage, the concentration of purified MS2 VLPs was determined using a Bradford assay (Pierce) with BSA serving as the protein standard.

MS2 expression and purification – method #2 (NYSBC)

The pCDF:MS2 plasmid was transformed into *E. coli* BL21(DE3) Gold cells (Agilent) and plated onto a Luria-Bertani (LB) agar plate containing 50 µg/mL streptomycin. A single colony was used to inoculate 20 mL of LB medium supplemented with the same antibiotic, and the culture was incubated overnight at 37°C with shaking at 225 rpm. This preculture was then used to inoculate 2 L of LB medium containing 50 µg/mL streptomycin and 0.01% Antifoam 204 and was grown at 37°C in a LEX bioreactor (Epiphyte Three Inc.) until an optical density of 0.8 measured at 600 nm was reached. Protein expression was induced by the addition of 1 mM isopropyl β-D-thiogalactopyranoside (IPTG) with overnight shaking at 25°C. Cells were harvested by centrifugation at 4,000*g* for 20 minutes and stored at −80°C until needed.

A cell pellet from 2 L of culture (approximately 9.5 g) was resuspended in 60 mL of 0.1 M potassium phosphate buffer pH 7.5 and lysed by sonication for 10 min (65% amplitude, 5 seconds on / 5 seconds off). Cell debris was removed by centrifugation at 27,000*g* for 15 minutes at 4°C. Ammonium sulfate was added to the supernatant to a final concentration of 0.265 g/mL and stirred until fully dissolved. The mixture was rotated for 1 hour at 4°C and precipitated proteins were collected by centrifugation at 27,000*g* for 15 minutes at 4°C. The resulting pellet was gently resuspended in 16 mL of 0.1 M potassium phosphate buffer pH 7.5 and incubated overnight at 4°C with gentle rotation. An equal volume of a 1:1 n-butanol/chloroform mixture was added, and the solution was vigorously mixed for 1 minute. After centrifugation at 27,000*g* for 10 minutes at 4°C, the upper aqueous phase was collected, diluted 50-fold with 10 mM Tris-HCl buffer pH 8.0, and filtered through a 0.4 µm filter. The clarified sample was loaded onto a Mono Q 10/100 GL anion exchange column (Cytiva) and MS2 protein was eluted using a NaCl gradient in 10 mM Tris(hydroxymethyl)aminomethane (Tris) pH 8.0. Fractions eluting around 500 mM NaCl were pooled, concentrated to 5 mL, and further purified using a HiLoad 16/60 Superdex 200 prep-grade column (Cytiva) equilibrated with 1x PBS pH 7.4. Purified protein fractions were pooled, concentrated to 10 mg/mL (measured by Bradford assay), and stored at −80°C.

bioRxiv preprint doi: https://doi.org/10.1101/2025.08.18.667046; this version posted August 18, 2025. The copyright holder for this preprint (which was not certified by peer review) is the author/funder, who has granted bioRxiv a license to display the preprint in perpetuity. It is made available under aCC-BY 4.0 International license.

MS2 capsid and MS2nc empty nanocrate cryo-EM data processing

Processing for empty nanocrates is summarized in **Figure S3**. Motion correction was performed with MotionCor2 to generate motion-corrected and dose-weighted images; all subsequent processing was done using cryoSPARC v4.6.2. CTF estimation was done using the Patch CTF module with default settings. Micrographs were curated based on a CTF resolution cutoff of 7 Å and a defocus range of 0.7–2.0 µm, resulting in 4,357 accepted and 443 rejected micrographs. Particles were picked using a single high-quality template view of MS2 with a particle diameter of 400 Å and 100 expected particles per micrograph. A total of 259,778 particles were extracted using a 480-pixel box size at a pixel size of 0.826 Å. The initial cleanup involved 2D classification with default settings, followed by the selection of sharp icosahedral classes (234,080 particles). *Ab initio* reconstruction and non-uniform refinement with enforced icosahedral symmetry, local CTF refinement, and EWS correction yielded a GSFSC 1.74 Å resolution map. The final resolution MS2nc dataset is the highest resolution of any MS2 structure reported to date, and the resulting density had resolved waters, so we proceeded to build an atomic model.

Coordinates from PDBID 2IZM with all non-protein atoms removed were used as an initial model. The asymmetric unit was rigid-body fitted into the map using UCSF Chimera. Iterative rounds of real space minimization and ADP refinement in Phenix[56] and manual inspection and adjustment in Coot[57] were performed. Only minor shifts in the rotamers of a few side chains were made from the initial model. Map peaks for water molecules were visible (**Fig S3E**). Waters were added to the model using the “Find Waters” feature in Coot for peaks above 2.0 σ. Added waters were curated by visual inspection, with additional waters added or deleted manually based on observed map peaks and the presence of neighboring hydrogen bonding partners. The final model includes an asymmetric unit of three chains, residues 1-129. Residues 1 and 13-15 of each chain are less resolved, but backbone density is visible. All other residues are well ordered with clear sidechain density (**Figure S3E**). The model includes 228 water molecules.

During this work, we generated a reconstruction of the MS2 capsid prior to the disassembly/reassembly process. The resulting reconstruction had equivalent map quality to the reassembled empty nanocrate. For completeness, we also built an atomic model into this map. The coordinates from the nanocrate model refinement, including the built waters, were used as a starting model. An initial round of rigid body, global real-space minimization, and ADP refinement was performed in Phenix. Minimal rotamer adjustments were made manually in Coot based on visual inspection, followed by iterative rounds of real space minimization and ADP refinement in Phenix and manual inspection and adjustment in Coot to improve the geometry and verify the placement of water molecules. Based on manual inspection, an additional ~40 waters were added and ~5 waters deleted relative to the nanocrate starting model. We did not analyze these differences in water structure further, as it is outside the scope of this work.

Cryo-EM maps and models were deposited to PDB/EMDB as follows:

bioRxiv preprint doi: https://doi.org/10.1101/2025.08.18.667046; this version posted August 18, 2025. The copyright holder for this preprint (which was not certified by peer review) is the author/funder, who has granted bioRxiv a license to display the preprint in perpetuity. It is made available under aCC-BY 4.0 International license.

9Q1B / EMD-72122	MS2 bacteriophage coat protein after reassembly as a nanocrate (MS2nc) with no cargo and with waters modeled
9Q1D / EMD-72124	MS2 bacteriophage coat protein with waters modeled
EMD-72123	Mouse apoferritin encapsulated in an MS2-nanocrate: MS2nc@ApoF
EMD-72176	Porcine thyroglobulin encapsulated in an MS2-nanocrate: MS2nc@Tg
EMD-72177	Dihydroneopterin aldolase encapsulated in an MS2-nanocrate: MS2nc@DHNA

MS2nc@ApoF cryo-EM data processing – particle subtraction and unsubtracted refinement

Processing of ApoF from nanocrates is summarized in **Figure S2,** and results are shown in **Figure S4**. Motion correction was performed using MotionCor2 to generate motion-corrected and dose-weighted images; all subsequent processing was carried out using cryoSPARC v4.6.2. CTF estimation was done using the Patch CTF module with default settings. Micrographs were curated based on a CTF resolution cutoff of 4.5 Å and a defocus range of 0.7–2.0 µm, resulting in 2,860 accepted and 2,036 rejected micrographs. Particles were picked using a single high-quality template view of MS2 with a particle diameter of 400 Å and 100 expected particles per micrograph. A total of 348,343 particles were extracted using a 480-pixel box size at a pixel size of 0.826 Å. Initial cleanup involved 2D classification with default settings, followed by selection of sharp icosahedral classes (239,460 particles). *Ab-initio* reconstruction and homogeneous refinement with enforced icosahedral symmetry, local CTF refinement and EWS correction produced a GSFSC 1.86 Å map. Particle subtraction was performed using particles from the final refinement. The resulting cage-subtracted stack was subjected to further 2D classification, and 69,412 particles containing ApoF were selected. ApoF-containing classes (~30% of the initial stack) were chosen for initial model generation and homogeneous refinement with octahedral symmetry enforced, resulting in a GSFSC 2.16 Å resolution map of ApoF (**Figure S4C**). Displayed at a lower threshold, we can see a noise shell around the ApoF, corresponding to the radially averaged out partially assembled MS2nc cage around ApoF molecules that can be seen in some 2D classes. Using the alignment information from the particle-subtracted reconstruction, we reconstructed particles from the unsubtracted stack without any refinement. The resulting map (**Figure S4E**) has achieved a GSFSC resolution of 2.19 Å. When displayed at the lower threshold,

bioRxiv preprint doi: https://doi.org/10.1101/2025.08.18.667046; this version posted August 18, 2025. The copyright holder for this preprint (which was not certified by peer review) is the author/funder, who has granted bioRxiv a license to display the preprint in perpetuity. It is made available under aCC-BY 4.0 International license.

a second noise shell can be seen around the ApoF density, corresponding to the MS2nc density that is radially averaged out.

MS2nc@Tg cryo-EM data processing – particle subtraction, local refinement, 3D classification and non-nanocrate processing

Processing of Tg from nanocrates is summarized in **Figure S5**. Motion correction was performed with MotionCor2 to generate motion-corrected and dose-weighted images; all subsequent processing was done using cryoSPARC v4.6.2. CTF estimation was done using the Patch CTF module with default settings. Micrographs were curated based on a CTF resolution cutoff of 4.0 Å and a defocus range of 0.7–2.0 µm, resulting in 7,549 accepted and 1,645 rejected micrographs. Particles were picked using a single high-quality template view of MS2nc with a particle diameter of 400 Å and 100 expected particles per micrograph. A total of 681,983 particles were extracted using a 480-pixel box size at a pixel size of 0.826 Å. Initial cleanup involved 2D classification with default settings, followed by selection of sharp icosahedral classes (541,146 particles). *Ab-initio* reconstruction and homogeneous refinement with enforced icosahedral symmetry yielded a GSFSC 2.02 Å map. After local CTF refinement and EWS correction, further homogeneous refinement improved the resolution to 1.92 Å. Particle subtraction was performed using particles from the final refinement. The resulting cage-subtracted stack was subjected to further 2D classification, and 131,048 particles containing Tg were selected. Homogeneous refinement with default settings and C2 symmetry resulted in a GSFSC 2.92 Å resolution map (**Figure S5C**). Symmetry expansion followed by masked local refinement of the asymmetric unit resulted in a GSFSC 2.80 Å resolution map, with a better-resolved distal part of the Tg map within the masked area (**Figure S5E**). At the same time, the distal part of the Tg map that was outside the mask became significantly distorted, suggesting that Tg molecules within the nanocrystals remained highly dynamic.

From the same dataset, we processed Tg particles that were not incorporated into nanocrates. These were picked using the template picker, 2D classified to retain only Tg-containing classes, further classified in 3D using *ab-initio* with 3 classes, and resulted in a final stack of 32,904 particles. Homogeneous refinement with C1 symmetry produced a GSFSC 3.87 Å resolution map, which was used to compare to the MS2nc@Tg derived map (**Figure 2A, 2B**).

MS2nc@DHNA cryo-EM processing –particle subtraction and custom symmetry expansion

Processing of DHNA from nanocrates is summarized in **Figure S6**. Motion correction was performed with MotionCor2 to generate motion-corrected and dose-weighted images, all subsequent processing was done using cryoSPARC v4.6.2. CTF estimation was done using the Patch CTF module with default settings. Micrographs were curated based on a CTF resolution cutoff of 4.5 Å and a defocus range of 0.7–2.0 µm, resulting in 6,360 accepted and 4,160 rejected micrographs. Particles were picked using a single high-quality template view of MS2 with a particle diameter of 400 Å and 100 expected particles per micrograph. A total of 252,070 particles were extracted using a 480-pixel box size at a pixel size of 0.826 Å. Initial cleanup involved 2D classification with default settings, followed by selection of sharp icosahedral

bioRxiv preprint doi: https://doi.org/10.1101/2025.08.18.667046; this version posted August 18, 2025. The copyright holder for this preprint (which was not certified by peer review) is the author/funder, who has granted bioRxiv a license to display the preprint in perpetuity. It is made available under aCC-BY 4.0 International license.

classes (149,058 particles). *Ab-initio* reconstruction and homogeneous refinement with enforced icosahedral symmetry yielded a 2.34 Å map. After local CTF refinement and EWS correction, further homogeneous refinement improved the resolution to 2.24 Å. Particle subtraction was performed using particles from the final refinement. The resulting cage-subtracted stack was subjected to further 2D classification, and 84,838 particles containing multiple DHNA copies were selected. A series of *ab-initio* classifications and homogeneous refinements led to a final subset of 25,543 particles displaying well-resolved DHNA packing (~10 Å resolution). Non-uniform refinement with a new reference and C5 symmetry enforcement yielded a GSFSC cargo map at 6 Å resolution. An additional non-uniform refinement with symmetry relaxation (marginalization) was applied to produce the final map used for sub-volume extraction. Local refinement was done following the cryoSPARC case study on encapsulated ferritin processing (see https://guide.cryosparc.com/processing-data/tutorials-and-case-studies/case-study-end-to-end-processing-of-encapsulated-ferritin-empiar-10716), beginning with five DHNA regions selected from the cargo map. Custom masks were used for sub-volume extraction with volume alignment tools in Chimera, enabling downstream local refinements and 3D classifications. Sub-volumes were aligned to a D4 symmetry axis, and 127,715 particles (5x expanded from the final cargo stack) were subjected to 3D classification into 10 classes. Selected particles (112,060) underwent local refinement with D4 symmetry, yielding a final map at 2.80 Å resolution.

Non-nanocrate DHNA cryo-EM data processing

Processing of DHNA from solution is summarized in **Figure S7**. Motion correction was performed with MotionCor2 to generate motion-corrected and dose-weighted images, all subsequent processing was done using cryoSPARC v4.6.2. CTF estimation was done using the Patch CTF module with default settings. Micrographs were curated based on a CTF resolution cutoff of 4.5 Å and a defocus range of 0.7–2.0 µm, resulting in 187 accepted and 211 rejected micrographs. Particles were manually selected to generate an initial template, and then the template picker was used with a particle diameter of 70 Å and an expected number of 4000 particles per micrograph. A total of 571,862 particles were extracted using a 256-pixel box size at a pixel size of 0.829 Å. Initial cleanup involved 2D classification with default settings, followed by selection of sharp, well-centered classes (218,565 particles). *Ab-initio* reconstruction and homogeneous refinement C1 symmetry produced a GSFSC 2.73 Å resolution highly anisotropic map that was used to compare with the nanocrate-derived DHNA map (**Figure 2C, 2D**). Homogeneous refinement with D4 symmetry also produced a highly anisotropic map that wasn't suitable for model building despite the GSFSC 2.29 Å resolution.

bioRxiv preprint doi: https://doi.org/10.1101/2025.08.18.667046; this version posted August 18, 2025. The copyright holder for this preprint (which was not certified by peer review) is the author/funder, who has granted bioRxiv a license to display the preprint in perpetuity. It is made available under aCC-BY 4.0 International license.

Supplemental Figures and Tables

Table S1. Protein compositions of reassembly mixtures for the MS2nc@ApoF, MS2nc@Tg, and MS2nc@DHNA samples presented in the main text.

Cargo Protein	MS2 monomer: cargo monomer ratio*	MS2 cage: cargo ratio**	MS2 monomer final conc. (μM)	Cargo monomer final conc. (μM)
ApoF (24-mer)	25:1	3.33:1	125	5
Tg (2-mer)	75:1	0.83:1	125	1.67
DHNA (8-mer)	12.5:1	0.56:1	125	10

* Ratio of MS2 coat protein monomer to cargo protein monomer

** Ratio of an assembled MS2 cage (180 CPs) to cargo in its quaternary state

Table S2. Example reassembly mixture.

Solution Component	Stock concentration	Final concentration	Volume added (μL)
MS2 solution (disassembled)	162 μM	125 μM	463.0
Apoferritin monomer	123 μM	5 μM	24.4
TMK buffer	10x	1x	60.0
Water	–	–	52.6
		Total volume	*600 μL*

bioRxiv preprint doi: https://doi.org/10.1101/2025.08.18.667046; this version posted August 18, 2025. The copyright holder for this preprint (which was not certified by peer review) is the author/funder, who has granted bioRxiv a license to display the preprint in perpetuity. It is made available under aCC-BY 4.0 International license.

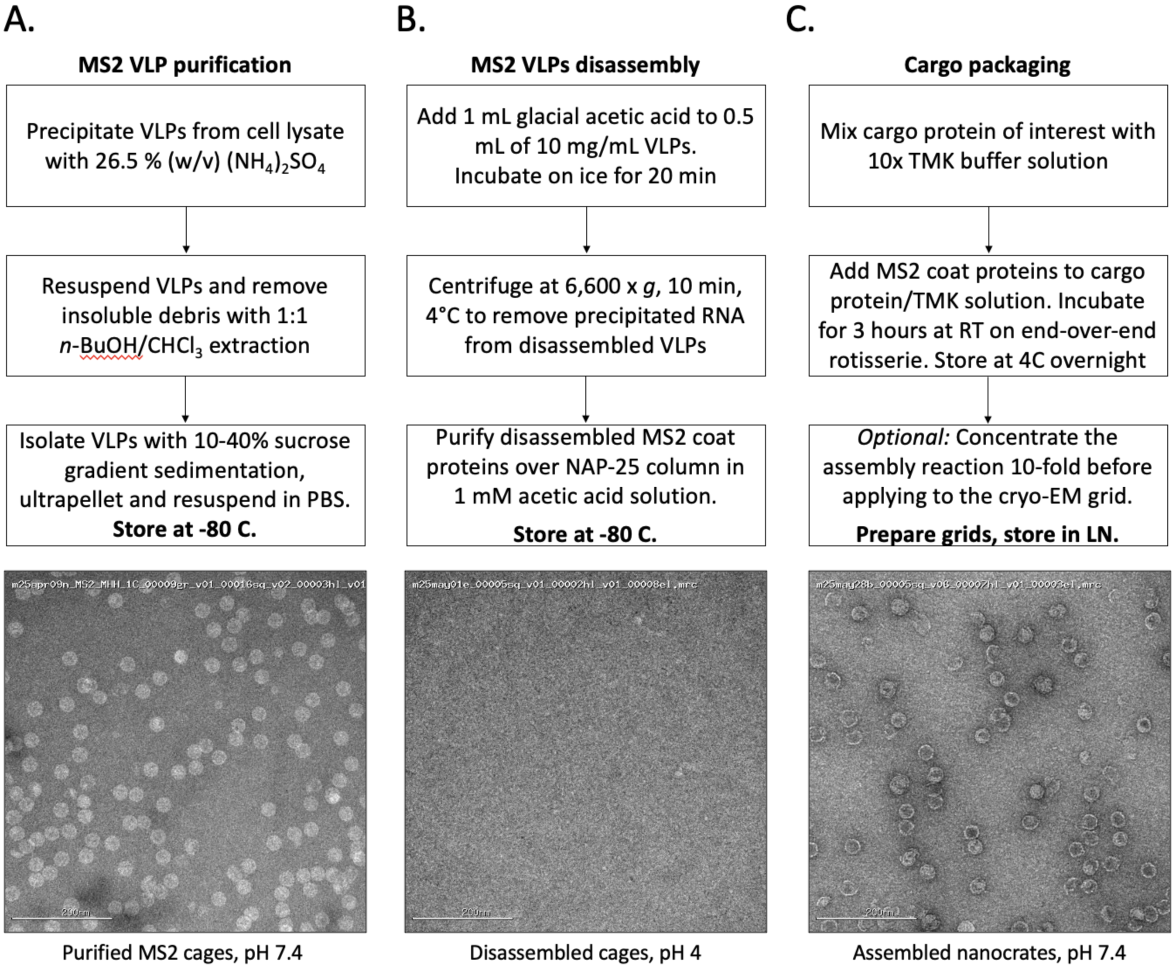

Figure S1. MS2 purification, disassembly and cargo packaging workflow.

bioRxiv preprint doi: https://doi.org/10.1101/2025.08.18.667046; this version posted August 18, 2025. The copyright holder for this preprint (which was not certified by peer review) is the author/funder, who has granted bioRxiv a license to display the preprint in perpetuity. It is made available under aCC-BY 4.0 International license.

Preprocessing	Data acquisition<br>**4,896 movies**	Leginon, pixel size 0.829 Å, total dose, 55 e^-/ Å^2, 50 frames
	Motion correction and dose weighting	Motioncor2: 7x5 tiles, global b-factor 500 Å^2, local b-factor 100 Å^2
	CTF estimation & curation<br>**2,860 exposures**	Defocus: 0.7-2.0 µm<br>CTF fit resolution: 2.2-7.0 Å<br>Relative ice thickness: 0.9-1.2 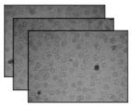
Nanocrate refinement	Particle picking and extraction<br>**348,343 particles**	Template picker: particle diameter 400 Å<br>Extraction: box 480 px 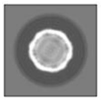
	2D classification and clean-up<br>**239,460 particles**	Accepted 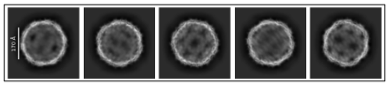<br>Rejected 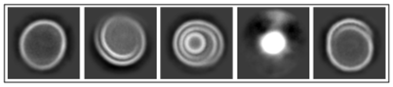
	Ab-initio (high symmetry)<br>**12 Å initial model**	Symmetry C1; 10,000 particles 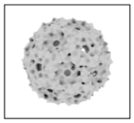
	Homogeneous Refinement<br>**GSFSC 1.86 Å nanocrate map**	Symmetry I; 239,460 particles; per-particle CTF, global CTF refinement, positive EWS correction 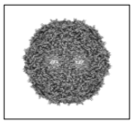
Cargo refinement (ApoF)	Particle subtraction, 2D classification and clean-up<br>**69,412 particles**	Accepted 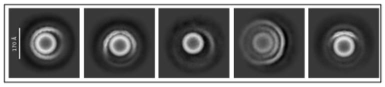<br>Rejected 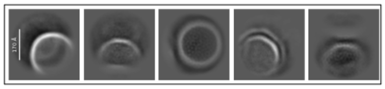
	Re-extract with recentering; ab-initio (high symmetry)<br>**12 Å initial model**	Symmetry C1; 10,000 particles<br>Extraction: box 480 px 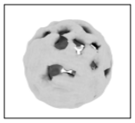
	Homogeneous Refinement<br>**GSFSC 2.15 Å cargo map**	Symmetry O; 69,412 particles; per-particle CTF 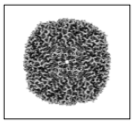

Figure S2. Caged particle reconstruction processing workflow.

bioRxiv preprint doi: https://doi.org/10.1101/2025.08.18.667046; this version posted August 18, 2025. The copyright holder for this preprint (which was not certified by peer review) is the author/funder, who has granted bioRxiv a license to display the preprint in perpetuity. It is made available under aCC-BY 4.0 International license.

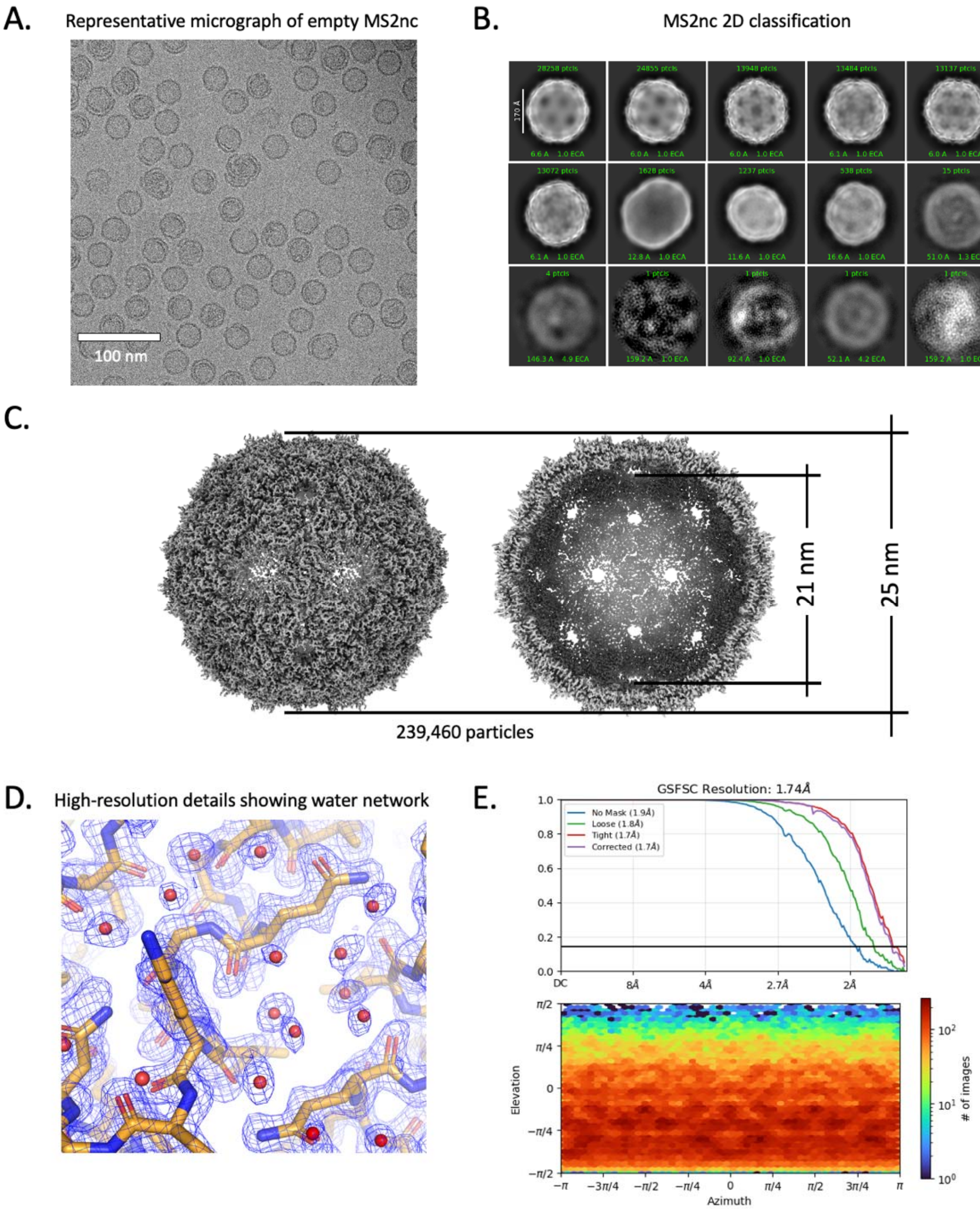

Figure S3. Empty MS2-nanocrate processing details. **A.** Representative micrograph of empty MS2-nanocrates. **B.** 2D classification of MS2nc without particle subtraction. **C.** refined map with I symmetry, MS2 nanocrate outer diameter = 25 nm, inner diameter = 21 nm, opening diameter of a 5-fold or 6-vertex pores = ~1.5 nm. **D.** Detailed view of a selected area of the MS-nanocrate map (shown as blue mesh), showing high-resolution resolvability of sidechains and coordinated water. Modeled protein is displayed in stick representation with carbon in tan, oxygen in red, and nitrogen in blue. Waters are depicted as red spheres. **E.** GSFSC plot showing 1.74 Å resolution and viewing direction distribution plot, showing isotropic orientation distribution.

bioRxiv preprint doi: https://doi.org/10.1101/2025.08.18.667046; this version posted August 18, 2025. The copyright holder for this preprint (which was not certified by peer review) is the author/funder, who has granted bioRxiv a license to display the preprint in perpetuity. It is made available under aCC-BY 4.0 International license.

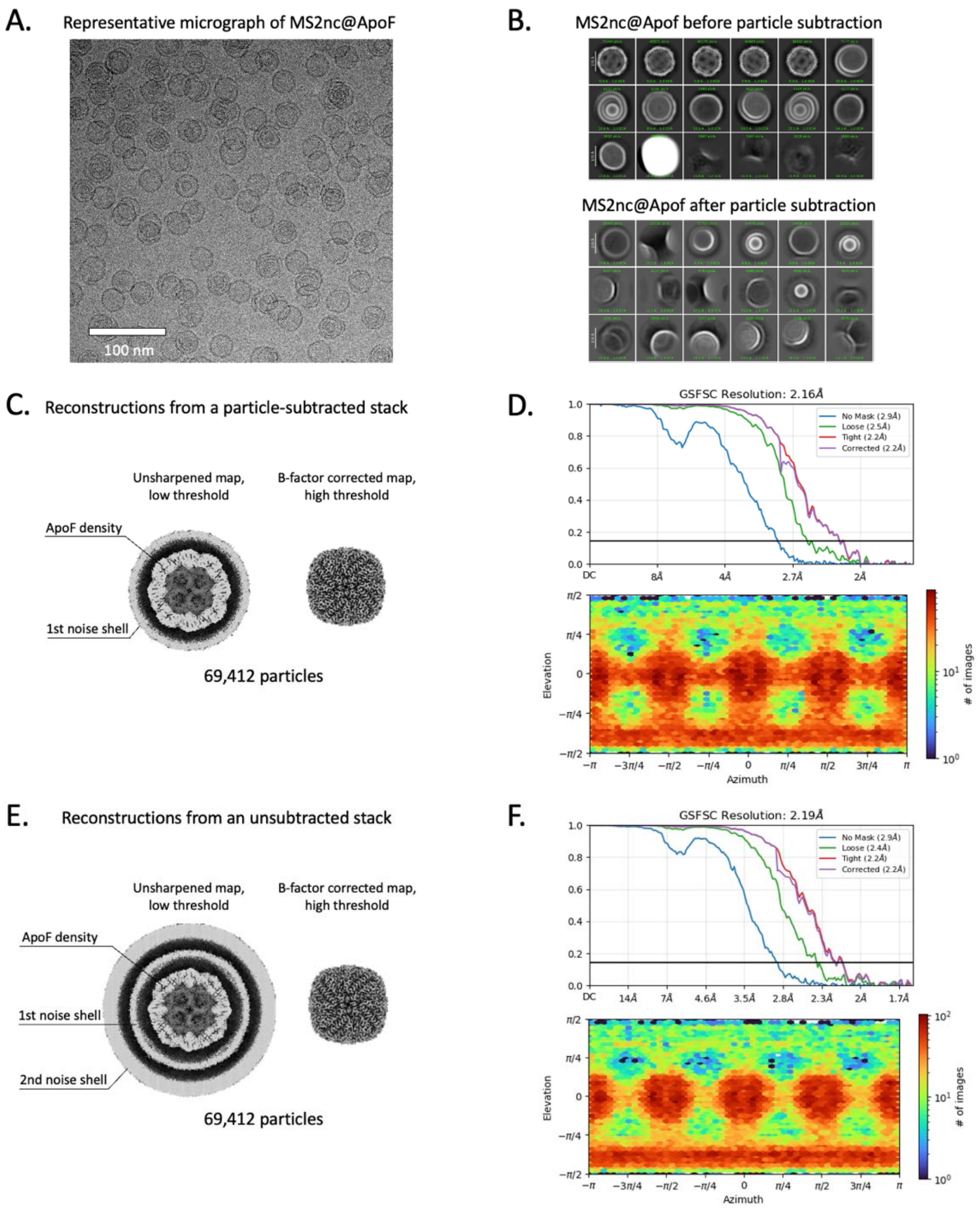

Figure S4. MS2nc@ApoF processing details. A. Representative micrograph. **B.** 2D classification before (top) and after nanocrate density subtraction. **C.** Refined map with O symmetry. **D.** GSFSC plot showing 2.16 Å resolution and viewing direction distribution plot, showing isotropic orientation distribution. **E.** Refinement with static mask, but without particle subtraction, using smaller box size and displayed at low threshold – nanocrate densities are averaged out into a uniform noise shell around the ApoF map. **F.** GSFSC plot showing 2.19 Å resolution and viewing direction distribution plot, showing isotropic orientation distribution.

bioRxiv preprint doi: https://doi.org/10.1101/2025.08.18.667046; this version posted August 18, 2025. The copyright holder for this preprint (which was not certified by peer review) is the author/funder, who has granted bioRxiv a license to display the preprint in perpetuity. It is made available under aCC-BY 4.0 International license.

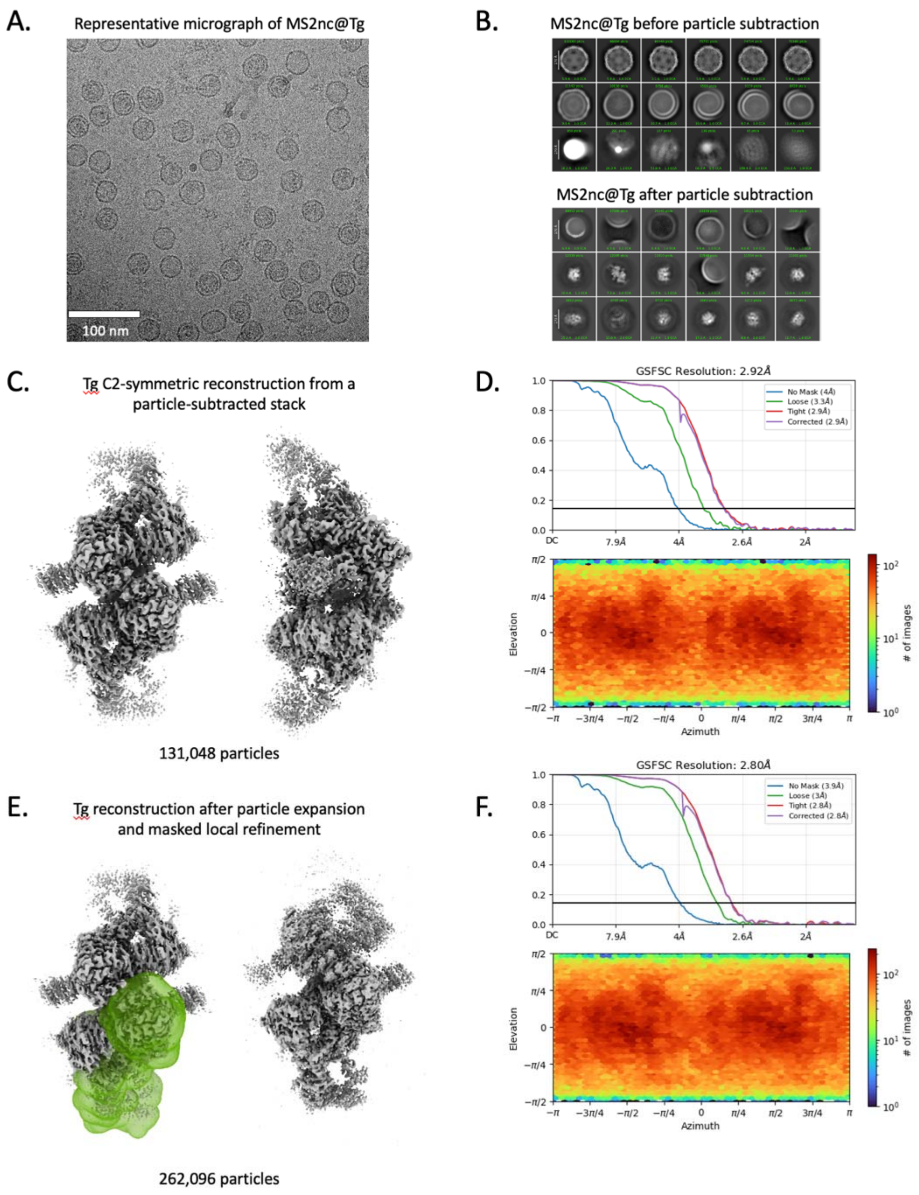

Figure S5. MS2nc@Tg processing details. **A.** Representative micrograph. **B.** 2D classification before (top) and after nanocrate density subtraction. **C.** Refined map with C2 symmetry. **D.** GSFSC plot showing 2.92 Å resolution and viewing direction distribution plot, showing isotropic orientation distribution. **E.** (*left*) Tg map with a mask region highlighted; (*right*) C1 reconstruction after local refinement of the masked region with corresponding GSFSC and viewing distribution plots **F**.

bioRxiv preprint doi: https://doi.org/10.1101/2025.08.18.667046; this version posted August 18, 2025. The copyright holder for this preprint (which was not certified by peer review) is the author/funder, who has granted bioRxiv a license to display the preprint in perpetuity. It is made available under aCC-BY 4.0 International license.

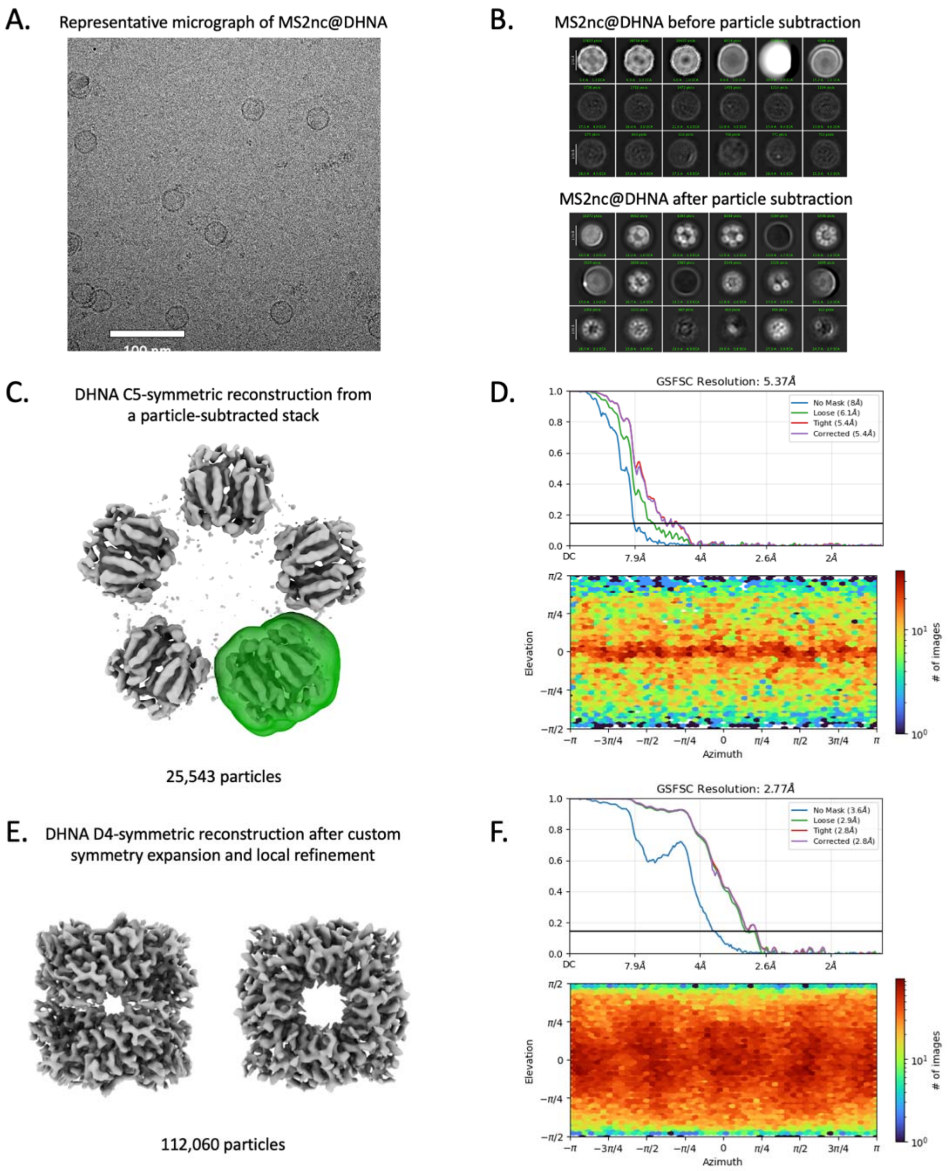

Figure S6. MS2nc@DHNA processing details. A. Representative micrograph. **C.** 2D classification before (*top*) and after nanocrate density subtraction. **C.** Refined map with C5 symmetry. A single DHNA copy was selected as a reference volume (masked in green) for symmetry expansion processing. **D.** GSFSC plot showing 5.375 Å resolution and viewing direction distribution plot, showing orientation distribution predominantly along the equator. **E.** Refined DHNA map after symmetry expansion and local refinement with D4 symmetry. **F.** GSFSC and viewing distribution plots corresponding to the reconstruction in **E**.

bioRxiv preprint doi: https://doi.org/10.1101/2025.08.18.667046; this version posted August 18, 2025. The copyright holder for this preprint (which was not certified by peer review) is the author/funder, who has granted bioRxiv a license to display the preprint in perpetuity. It is made available under aCC-BY 4.0 International license.

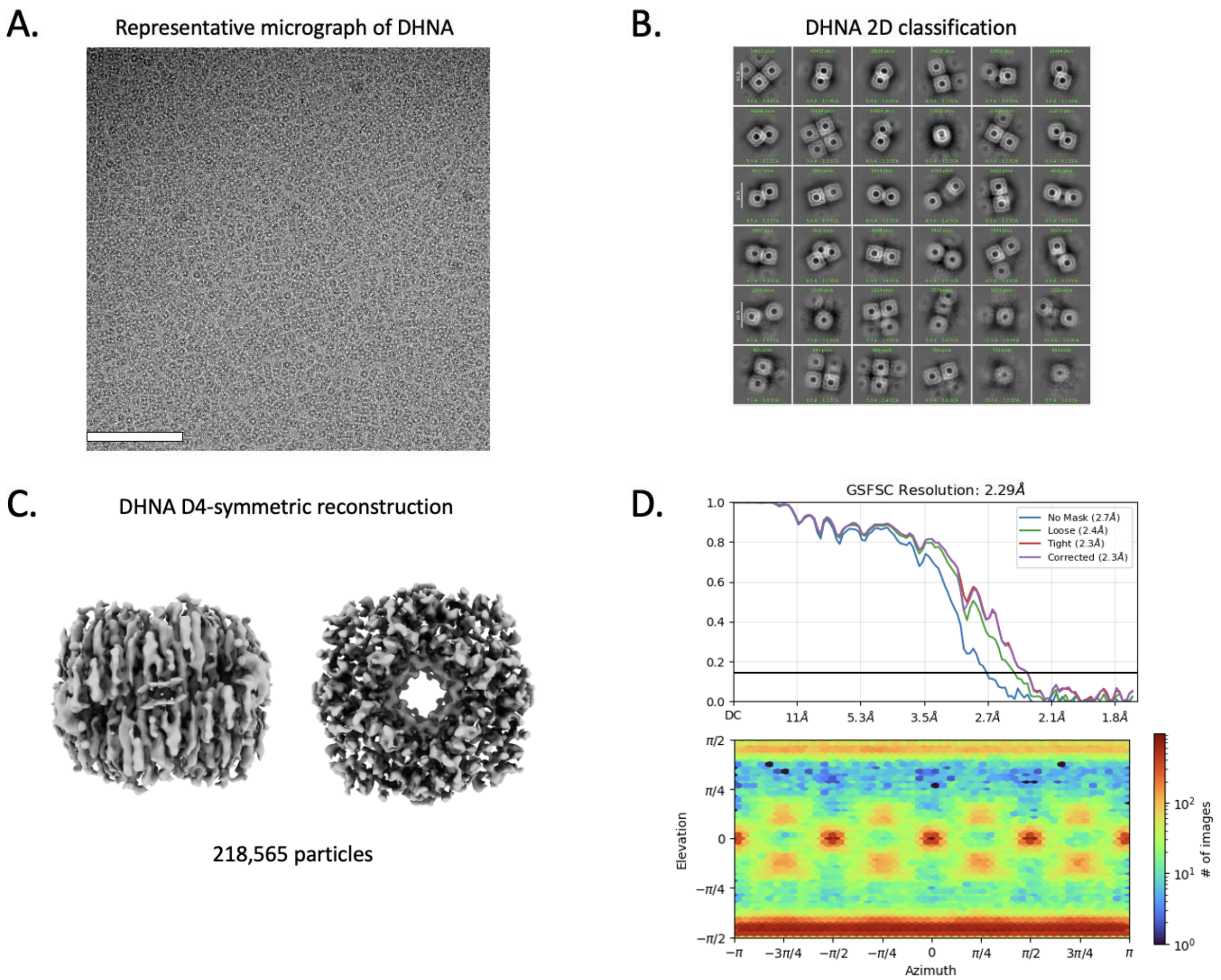

Figure S7. DHNA processing without nanocrates. A. Representative micrograph. **B.** 2D classification showing exclusively top views. **C.** Refined map with D4 symmetry enforced. The map is highly anisotropic and stretched in the direction of the preferred view, making it unsuitable for model building **D.** GSFSC plot showing 2.29 Å resolution and viewing direction distribution plot, showing highly preferred orientation.

bioRxiv preprint doi: https://doi.org/10.1101/2025.08.18.667046; this version posted August 18, 2025. The copyright holder for this preprint (which was not certified by peer review) is the author/funder, who has granted bioRxiv a license to display the preprint in perpetuity. It is made available under aCC-BY 4.0 International license.

Cryo-EM data collection, refinement, and validation statistics for MS2 capsid and empty MS2nc

	MS2 capsid (EMD-72124) (PDB 9Q1D)	MS2nc no cargo (EMD-72122) (PDB 9Q1B)
**Data collection and processing**		
Magnification	105,000	105,000
Voltage (kV)	300	300
Electron exposure (e–/Å^2)	52.36	54.58
Defocus range (μm)	-0.7 to -2.0	-0.7 to -2.0
Pixel size (Å)	0.829	0.829
Symmetry imposed	I	I
Particle images (no.)	106,754	239,460
Map resolution (Å)	1.75	1.74
FSC threshold	0.143	0.143
**Refinement**		
Initial model used (PDB code)	9Q1B	2IZM
Model resolution (Å)	1.75	1.74
FSC threshold	0.143	0.143
Map sharpening *B* factor (Å^2)	45.4	47.6
Model composition		
Non-hydrogen atoms	3160	3123
Protein residues	387	387
Waters	265	228
Map-to-model FSC at threshold of 0.5		
CCvolume/CCmask		
B factors (Å^2) (min/max/mean)		
Protein	6.38/85.57/21.79	1.34/68.84/11.65
Waters	15.54/60.71/31.30	4.92/39.09/18.55
R.m.s. deviations		
Bond lengths (Å)	0.003	0.002
Bond angles (°)	0.543	0.494
Validation		
MolProbity score	1.04	1.06
Clashscore	2.59	2.76
Poor rotamers (%)	0.00	0.00
Ramachandran plot		
Favored (%)	98.69	98.95
Allowed (%)	1.31	1.05
Disallowed (%)	0.00	0.00
CaBLAM outliers (%)		
EMRinger score		
Average Q-score	0.789	0.80
